# The Clinical Value of the Gonorrhea Complement Fixation Test

**Published:** 1913-04

**Authors:** 


THE CLINICAL VALUE OF THE GONORRHEA COMPLEMENT FIXATION TEST. Victor D. Lespinasse, M. D., and Maurice Wolff, M. D., Chicago. *Illinois Medical Journal*, Jan., 1913—These antibodies persist in the blood as long as there are any live gonococci present in the body; just how long they remain in the bloodstream after the death of the last gonococcus is not definitely known, but they probably do not persist very long. We have some work in progress at present to determine this point. In our series of cases of individuals with gonorrheal history, cited below, there is not a single positive reaction occurring after two years. This fact bears out Keyes' work in the persistency of the gonococcus in the urethera. According to the conclusion of Keyes, the gonococcus rarely if ever persists in the urethra longer than 18 months. According to the gonorrhea complement fixation test, the antibodies can be present at least for two years.

The 25 cases headed "No gonorrheal history" are children and individuals who can be relied on to give honest statements.

| Case. | No. of Cases. | Positives. | Pct. | Negatives. | Pct. |
|---|---|---|---|---|---|
| No gonorrheal history..... | 25 | 0 | 0 | 25 | 100 |
| All the following cases were clinically well with the following gonorrheal history: | | | | | |
| 1 year.................. | 12 | 3 | 25 | 9 | 75 |
| 2 years.................. | 9 | 1 | 11 | 8 | 89 |
| 3 years.................. | 8 | | | | |
| 4 years.................. | 2 | | | | |
| 5 years.................. | 10 | | | | |
| 6 years.................. | 2 | | | | |
| 7 years.................. | 3 | | | | |
| 8 years.................. | 6 | | | | |
| 9 years.................. | 4 | | | | |
| 10 years.................. | 12 | | | | |
| All negatives. | | | | | All negatives. |

In contra-distinction to the Wassermann test, which will become negative with mercurial or salvarsan treatment, there is no treatment that will render the gonorrhea complement fixation test negative. But there is a method of treatment that will render any one positive to the gonorrhea complement fixation test, whether he has a gonorrhea or not, namely: the injection of gonorrhea vaccines. Therefore, in cases recently treated with gonococcic vaccines, the gonorrhea complement fixation test is of little value.

